# A preliminary model to establish a digital twin for coffee roasting

**DOI:** 10.1038/s41598-026-43923-9

**Published:** 2026-04-30

**Authors:** Manuel Jonathan Bruno, Nadaniela Egidi, Lorella Fatone, Josephin Giacomini, Pierluigi Maponi, Gianni Sagratini, Agnese Santanatoglia, Edin Trebović

**Affiliations:** 1 School of Science and Technologies, Mathematics Division, Università degli studi di Camerino, Via Madonna delle Carceri 9, 62032 Camerino, Italy; 2 Chemistry Interdisciplinary Project (CHIP), School of Pharmacy, Università degli studi di Camerino, Via Madonna delle Carceri 9/B, 62032 Camerino, Italy; 3 Research and Innovation Coffee Hub (RICH), Via Emilio Betti 1, 62020 Belforte del Chienti, Italy; 4 Gambilongo Caffè srl, Via dell’Artigianato 6 - Z.I. Montalto Uffugo, 87046 Rende, Italy

**Keywords:** Coffee, Roasting, Kinetics, Optimisation, Chemistry, Engineering, Mathematics and computing

## Abstract

The coffee industry has an economic impact of over 100 billion dollars per year, making it one of the most valuable markets in the world. Coffee roasting is a crucial step that occurs before the extraction process and is essential to the final quality of the coffee. The roasting of green coffee beans is a complex process involving various chemical reactions that play a crucial role in determining the taste, colour and aroma of the coffee. It consists of three steps: drying, roasting and cooling. All of them are characterised by heat and mass transfer. Heat transfer during the roasting process significantly influences the flavour profile of a coffee cup. The main chemical reaction that occurs during roasting is known as the “Maillard reaction”, which is fundamental for the sensory profile of roasted coffee. In this study, we first introduce a mathematical model for coffee roasting based on the chemical dynamics of key compounds. The kinetic model is used to determine the variation in concentration of the main chemical substances that characterise the taste and aroma of coffee. The calibration of the model is obtained through an optimisation procedure, capable of estimating the kinetic rate constants. Real data from chemical analyses carried out on coffee samples at the end of the roasting process were used to support the calibration phase, while the initial chemical composition of green coffee was obtained from the ranges available in the literature.

## Introduction

Coffee is one of the most popular commodities worldwide^50^. The intake of this drink approaches about two billion cups per day, making it the most widely consumed beverage after water and tea^67^. Coffee consumers and, consequently, the way in which this beverage is enjoyed vary considerably depending on factors such as age, culture and geographical location. What makes this beverage so appealing is undoubtedly the aroma released during the extraction phase, which results from the presence of over 1000 compounds found in coffee^4^.

The aroma is certainly not the only explanation for coffee’s popularity; in fact, the presence of nutrients essential for human health, such as lipids, proteins, carbohydrates, and minerals like potassium and magnesium, also plays a significant role. One of the main benefits of coffee is its high antioxidant content, natural compounds that help fight free radicals, which are responsible for cellular ageing and many diseases. The antioxidants found in coffee, such as polyphenols and chlorogenic acid, protect the body’s cells and support the immune system^5^. The presence of caffeine, the main active compound in coffee, provides a stimulating effect on the central nervous system. This effect can improve concentration, attention and cognitive performance. Numerous studies suggest that moderate coffee consumption may help control movement disorders associated to Parkinson’s disease^51^, as well as having anti-inflammatory^60^ and anti-carcinogenic properties^66^. Obviously, these beneficial effects are observed with moderate consumption of the beverage, otherwise they would turn into negative effects such as heart palpitations, insomnia and hyper-excitability^18^.

Research on coffee is important from both a nutritional and an industrial point of view. In particular, preparing coffee in a customised way is one of the next goals in the coffee sector^45^. Personal tastes are influenced by cultural and environmental factors, as well as health issues and temporary needs. For example, a cup with a higher caffeine content may be preferred during a long drive, whereas a lighter drink may be better after a heavy meal. When it comes to coffee, smell is the first sense involved, but immediately afterwards, and with no small effect, taste plays a major role. A wide variety of flavours can be evoked when drinking coffee, such as sour, nutty, sweet, tart, fruity, roasted and caramel. These flavours are associated with specific chemical compounds that are released from the coffee powder during the extraction process and end up in the cup of coffee^2,7^. From beans to cup, the final quality of coffee is the result of an integrated sequence of stages. First, there are cultivation and harvesting, processing of the drupe, drying and storage of green coffee^37^. Next, come the physicochemical processing stages, namely roasting, grinding and finally extraction with hot water. The first process that green coffee beans undergo is roasting. In some ways, this stage creates the bean’s signature, changing its colour, nutritional content and aromatic profile^62^, before grinding and percolation^3^, complete the transformation of the beverage.

Roasting is a heat treatment used not only on coffee beans but also on other foods such as cocoa and hazelnuts^58^. This process occurs at high temperatures, typically between $150^\circ$C and $250^\circ$C. During this operation, the beans lose most of their water content, tend to expand and undergo significant physical and chemical changes. There are various roasting methods, including drum roasting and hot air roasting. In any case, time and temperature are the key variables that determine the final profile of the beverage^42^. Roasting is a crucial step, as it defines the taste and aroma of the coffee, improves digestibility, and also contributes to the differentiation of the product on the market. Several chemical reactions occur during this phase, and they are essential for the development of the taste and colour of roasted coffee, namely thermal degradation, caramelisation, Maillard and Strecker reactions^9^.

At temperatures of $150-170^\circ$C, the carbonyl groups derived from sugars and the amino acids that comprise proteins react to form the key compounds responsible for the aroma and taste of the beverage. Hundreds of chemical compounds that influence the final taste are produced by Maillard reactions, including melanoidins^35^. Starting at temperatures of $170-200^\circ$C, the sugars in coffee begin to caramelise, turning brown and releasing aromatic compounds. During roasting, most of the sucrose is converted into other products^49^. At around $205^\circ$C, the heat absorbed by convection or conduction, depending on the method used, causes the bean to expand and produces the so-called first crack^56^. At this point, the colour of the beans changes from green/yellow to light brown. Additionally, the bean loses approximately 5% of its initial weight due to water evaporation. Once the first crack has ended, the roasting process continues, with the beans gradually darkening. Later, in the medium roast stage, as the temperature in the bean reaches around $220^\circ$C, the beans undergo a chemical reaction known as pyrolysis (thermal degradation), during which carbon dioxide (and other volatile compounds) is released from the bean’s structure^27^. This modulates the acidic flavours and produces a more rounded and integrated flavour within the bean. The beans also lose additional weight at this point (around 13%), as the carbon dioxide is gradually expelled. At the temperature of $230^\circ$C, the beans experience the so-called second crack^56^. In this phase, the bean is visibly characterised by colours ranging from dark brown to almost black. It also becomes shiny, as oil from inside migrates to the surface, coating the beans and giving them a glossy appearance^25^. Once the coffee has been heated to the desired roast level, the beans are removed from the roaster and cooled to halt the roasting process.

Experimental and sensory chemical analyses of roasted coffee can be combined with the development of mathematical models capable of describing and predicting the transformations that occur during roasting as a consequence of simultaneous heat and mass transfer and complex chemical reactions, which determine the evolution of the physical, chemical and organoleptic properties of the bean. Accurate models can support the design of a digital twin for the optimisation of industrial roasting processes by acting as a link between operating conditions, i.e., temperature, airflow, and roasting time, and the final composition and quality parameters of the product, such as colour, acidity, and aroma precursors. In addition, such prediction tools can also support the customisation of coffee preparation by providing important information, i.e., the chemical characterisation of the roasted coffee without the need for its chemical analysis. To this end, kinetic modelling has been extensively applied in the field of food processing to describe the evolution of chemical, physical and biochemical transformations occurring during thermal treatments. Kinetic approaches based on ordinary differential equations have been widely used to model degradation, formation and conversion reactions of key food constituents, such as nutrients, bioactive compounds, colour-related molecules and aroma precursors. These models provide a quantitative link between processing conditions, especially time and temperature, and the resulting product composition, enabling process optimisation, quality control and prediction of product behaviour under different operating scenarios. In recent years, the integration of kinetic modelling with experimental data has also supported the development of digital and predictive tools for food processing systems. A detailed description of such ideas can be found in Göncuüoğlu Tşs and Gökmen^30^; Şen and Gökmen^68^; Lee et al.^39^. However, other approaches have been used to analyse the internal transformations of the coffee bean; in this context, ^6,21,22^ describe the phenomena of mass and heat transfer within the bean. These are very often based on convective-diffusive partial differential equations and require careful study of the bean’s geometry.

In this work, we develop a chemical-kinetic model aimed at reproducing the evolution of the concentration of the most important chemical substances during roasting. This model is fundamental for the development of a digital twin for coffee customisation, together with another important mathematical model describing the coffee percolation process^3,15,16,26^, which we are currently developing. In particular, four roasted coffee powders from distinct geographical origins, Mexico and Rwanda (*Coffea arabica L.*), Nicaragua and Indonesia (*Coffea canephora Pierre ex A. Froehner*), were analysed. This preliminary model takes into account the main compounds that play a crucial role in the final profile of the beverage. A system of ordinary differential equations governs the dynamics of the substance concentrations. In the proposed model, we assume that each considered compound undergoes a first (or second) degradation or transformation reaction, and the parameters that determine the reaction rates are calibrated through an optimisation procedure. At the moment, the objective function is constructed by using the laboratory measurements of the most important chemical substances. This approach enables the quantification of the rates of degradation and formation of key compounds during roasting, highlighting the complex interaction between the loss of precursors and the generation of secondary products.

Ultimately, the main strengths of this work are the following.

- Compared to the roasting models found in the literature, such as Fadai et al.^22^ or Şen and Gökmen^68^, here we provide a clear definition and implementation of a mathematical model capable of describing the evolution of the key chemicals that give coffee its taste and aroma.
- The developed mathematical model paves the way for the creation of a tool able to meet the needs and requirements of the end consumer, and therefore for product customisation. This aspect is obviously closely linked to the concept of digital twins.
- The proposed method translates the chemical-kinetic rules given by the literature into first-order and second-order reactions, where the rate constants are assumed to follow the Arrhenius law. The chemical-kinetic model is mass-preserving and depends on unknown kinetic parameters. Hence, the proposed model needs to be calibrated. This calibration uses literature data on initial conditions, final laboratory measurements of some chemical compounds considered in the model and some temperature measurements during roasting. Despite the limited laboratory data available to us, the calibrated model provides qualitatively good results.

The paper is organised as follows. Section “Experimental analyses” provides all the information on how the chemical analyses were obtained on the compounds of interest within the coffee bean at the end of the roasting phase. Section "Behaviour of chemical compounds during roasting and modelling assumptions" defines the various precursor-product relationships between the substances of interest, in order to appropriately justify the chemical network that has been hypothesised. Section “Kinetic modelling” introduces the basic mathematical language that allows to describe the kinetics of chemical reactions. Section "The mathematical model" introduces the network of chemical reactions involved in coffee roasting, formulates the mathematical model describing the temporal evolution of the concentrations of the chemical compounds, and develops a calibration procedure for estimating the reaction rate constants. Implementation details are also provided in this section. Section "Results and discussion" is dedicated to the results obtained from the mathematical model. Finally, Section “Conclusion” provides conclusions and information on future developments.

## Experimental analyses

We describe the experimental work aimed at quantifying the main chemical constituents of roasted coffee beans, which are directly involved in the roasting kinetics. In detail, caffeine, trigonelline, chlorogenic and phenolic acids, lipids and major organic acids were analysed in coffee samples from four different geographical origins. The chemical analyses were performed using validated chromatographic and extraction procedures to provide reliable concentration data for the development and calibration of the kinetic model described in this study.

### Chemicals and reagents

Organic acids, namely, tartaric acid ($\ge$99.5%, C_4_H_6_O_6_, CAS No 87-69-4), formic acid (solution for HPLC, 99%, CH_2_O_2_, CAS No 64-18-6), acetic acid (solution for HPLC, C_2_H_4_O_2_, CAS No 64-19-7), citric acid ($\ge$99.5%, C_6_H_8_O_7_, CAS No 77-92-9) were purchased from Sigma Aldrich (St. Louis, MO, USA). Deionised water was purified using a Milli-Q SP Reagent Water System (Millipore, Bedford, MA, USA). LC-MS grade methanol and formic acid were supplied by Sigma-Aldrich (Milano, Italy). HCl (37%, HCl, CAS No 7647-01-0) was supplied by Carlo Erba reagents, while NaOH ($\ge$98%, NaOH, CAS No 1310-73-2) was provided by Sigma-Aldrich (St. Louis, MO, USA). The solution of phosphoric acid (85%, H_3_PO_4_, CAS No 7664-38-2) and the ammonium hydroxide solution (30–33%, CAS No 1336-21-6) were provided by Sigma-Aldrich (St. Louis, MO, USA). Potassium dihydrogen phosphate (99.9%, KH_2_PO_4_, CAS No 7778-77-0) was supplied by Carlo Erba reagents. The analytical standards of 5-O-caffeoylquinic acid (5-CGA, $\ge$98%, C_16_H_18_O_9_, CAS No 906-33-2), 3-O-caffeoylquinic acid (3-CGA, $\ge$98%, C_16_H_18_O_9_, CAS No 327-97-9) and 3,5-dicaffeoylquinic acid (3,5-diCGA, $\ge$98%, C_25_H_24_O_12_, CAS No 2450-53-5), caffeine ($\ge$99%, C_8_H_10_N_4_O_2_, CAS No 58-08-2) were purchased from Sigma-Aldrich (Milano, Italy) and trigonelline ($\ge$99%, C_7_H_7_NO_2_, CAS No 535-83-1).

### Coffee samples

Roasted coffee beans from four geographical origins - Mexico and Rwanda (Coffea arabica L.) and Nicaragua and Indonesia (Coffea canephora Pierre ex A. Froehner) - were used for the analyses. The first two single-origin coffee samples were medium-roasted using a drum roaster (IMF), with a drum speed ranging from 81% to 92%, an airflow temperature in the whole process from $220^\circ$C to $340^\circ$C, a bean temperature endpoint of $212\pm 2^\circ$C and a total roasting time of 12–13 min. These conditions correspond to a medium roast profile. On the other hand, Nicaragua and Indonesia coffees were hard-roasted using a drum roaster (IMF), with a drum speed ranging from 88% to 94%, an airflow temperature in the whole process from $220^\circ$C to $395^\circ$C, a bean temperature endpoint of $229 \pm 1^\circ$C and a total roasting time of 14–15 min. All samples were stored under refrigeration (-$15^\circ$C). Grinding was carried out using a Mythos 1 grinder (Nuova Simonelli, Italy). The optimal grind setting was calibrated to yield $40 \pm 2$ g of brewed coffee from $20 \pm 0.1$ g of ground coffee, using a VST Competition filter (20 g basket) and applying a tamping force of 20 kgF with a PuqPress M2 automatic tamper. For each coffee origin, three grind sizes: optimal, finer and coarser, were prepared, immediately transferred into containers, and stored at -$15^\circ$C until analysis. For chemical and granulometric determinations, aliquots of the three grind sizes (optimal, finer and coarser) were combined in equal mass proportions (1:1:1, *w*/*w*/*w*) to obtain a representative composite sample for each origin. This compositing strategy was adopted to reduce the influence of grind-size heterogeneity on chemical composition, to approximate the average particle size distribution encountered in practical espresso preparation. The adoption of a composite sampling strategy based on the combination of different grind sizes was motivated by the need to obtain a representative sample of ground coffee, minimising the effect of particle size heterogeneity on chemical analyses. This approach is commonly used in coffee research, as it allows the reduction of sampling bias associated with segregation phenomena and grind-size-dependent extraction behaviour. By averaging fine, medium and coarse fractions in equal proportions, the resulting composite sample better reflects the overall composition of the roasted coffee batch rather than a specific granulometric class.

### Particle size analysis

Particle size distribution of the ground coffee samples was determined using a Mastersizer 3000 Aero Series laser diffraction analyser (Malvern PANalytical Ltd., Malvern, UK) equipped with a dry dispersion unit. The instrument measurements particle size distributions over the range of 0.01–3500 $\upmu$m by detecting the angular scattering of laser light by dispersed particles. Dry dispersion was performed under continuous airflow generated by a mechanical compressor operating at 6.5 bar, while the powder was conveyed to the laser measurement zone at a dispersion pressure of 2 - 3 bar. Each sample was measured five times, and the mean distribution values were used for comparisons among coffee origins. The average median particle diameter ($\text {D}_{50}$) of the composite samples was approximately $270 \pm 15 \upmu$m, consistent with a medium espresso grind.

### Caffeine, trigonelline, chlorogenic and phenolic acids (ferulic acid) analysis

Briefly, 0.2 g of ground coffee powder from each origin (Indonesia, Nicaragua, Mexico and Rwanda) was extracted with 10 mL of a methanol: water (50:50, *v/v*) solution in an ultrasonic bath for 30 min. The obtained extracts were centrifuged at 4,500 rpm for 10 min and subsequently filtered through a 0.22 $\upmu$m PTFE membrane filter before HPLC-DAD analysis. HPLC-DAD analysis of caffeine, trigonelline, chlorogenic and phenolic acids was performed according to the method previously developed by Santanatoglia et al.^53,54^. The chromatographic system consisted of an Agilent 1100 series (Agilent Technologies, Santa Clara, CA, USA) equipped with a diode array detector (DAD), binary pump and autosampler. The Injection volume was 3 $\upmu$L, and separation was achieved using a Gemini C18 analytical column (250 mm $\times$ 3.0 mm, 5 $\upmu$m) preceded by a SecurityGuard C18 guard column (4 cm $\times$ 3 mm, 5 $\upmu$m) (Phenomenex, Torrance, CA, USA). The column temperature was maintained at $40^\circ$C and elution was performed in gradient mode using water (A) and methanol (B), both containing 0.1% formic acid, as the mobile phases. The gradient consisted of: 0–10 min: 20% B (isocratic); 10–15 min: linear increase to 35% B; 15–20 min: linear increase to 55% B; 20–25 min: linear increase to 85% B; 25–30 min: return to 20% B; 30–35 min: re-equilibration at 20% B. The flow rate was 0.8 mL/min, and detection was performed at 325 nm for chlorogenic and phenolic acids, and 270 nm for caffeine and trigonelline. Quantification was carried out by external calibration using standard solutions of each analyte ($\ge$99% purity, Sigma-Aldrich, St. Louis, MO, USA).

### Organic acids analysis (citric, tartaric, acetic)

Briefly, approximately 1.0 g of ground coffee powder from Indonesia, Nicaragua, Mexico and Rwanda was weighed into centrifuge tubes and extracted with 10 mL of ultrapure Milli-Q water. The suspensions were vortex-mixed for 1 min and centrifuged at 5,000 rpm for 5 min. The supernatant was collected, and its pH was adjusted to 8 using a 0.1 M NaOH solution, then filtered through a 0.45 $\upmu$m membrane filter. For the purification step, a Strata-X SPE cartridge (200 mg/6 mL polymeric reversed phase, Phenomenex, Bologna, Italy) was used according to the method previously published by Santanatoglia et al.^54^. Before analyte purification, cartridges were activated with 1 mL of methanol, and conditioned with 1 mL of ultrapure water and 2 mL of 33% ammonia solution. Then, 0.6 mL of the filtered extract, previously adjusted to pH 7–8, was loaded onto the cartridge and washed with 1 mL of 33% ammonia solution (applied as two 0.5 mL portions). Finally, 4 mL of 1 M HCl was used for elution, dispensed as eight 0.5 mL fractions. The eluates were collected into clean glass tubes for subsequent instrumental analysis. HPLC-DAD analysis was performed using an Agilent 1100 series system (Agilent Technologies, Santa Clara, CA, USA) equipped with a diode array detector (DAD), binary pump and autosampler. The injection volume was 20 $\upmu$L, and a Luna Omega Polar C18 column (250 mm $\times$ 4.6 mm, 3 $\upmu$m) preceded by a SecurityGuard C18 guard column (4 cm $\times$ 3 mm, 5 $\upmu$m) (Phenomenex, Torrance, CA, USA) was employed for separation. The column temperature was maintained at $30^\circ$C and elution was performed in isocratic mode using a 0.1 M phosphate buffer (KH_2_PO_4_ adjusted to pH 2.5 with 0.85% phosphoric acid) at a flow rate of 0.8 mL/min. Detection was carried out at 210 nm for tartaric, acetic and citric acids. Quantification was achieved by external calibration using standard solutions of each organic acid ($\ge$99% purity, Sigma-Aldrich, St. Louis, MO, USA).

### Lipid extraction

Roasted coffee powders from Rwanda, Nicaragua, Indonesia and Mexico were used for lipid extraction. The procedure was performed according to the method described by Hibbert et al.^34^, with slight modifications. Approximately 5.0 g of ground coffee were placed in a cellulose extraction thimble and subjected to Soxhlet extraction (Universal Extractor - Buchi, Mod. E-800, Uster, Switzerland) using 260 mL of cyclohexane as solvent. The extraction process was carried out for 4 h under continuous reflux conditions. After extraction, the solvent was removed under reduced pressure using a rotary evaporator (Rotavapor) until complete dryness. The residue obtained was weighed to determine the total lipid content on a dry-weight basis ($\%\,w/w$), which was calculated according to the following formula

$$\begin{aligned} \text {Total lipids (}\%\,w/w\mathrm{)} = \dfrac{m_1-m_0}{m_s} \times 100, \end{aligned}$$

where $m_1$ is the mass of the flask after solvent evaporation, $m_0$ is the mass of the empty flask, and $m_s$ is the mass of the coffee sample used for extraction.

## Behaviour of chemical compounds during roasting and modelling assumptions

The enjoyable flavour of coffee is mainly attributed to a variety of biochemical compounds. These components depend on several factors, including botanical species, geographical origin, post-harvest processing and roasting conditions, all of which can influence the concentration of both volatile and non-volatile components^29^. In every variety of green coffee, from Arabica to Robusta, we can find different components^19^, for instance, methylxanthines (caffeine, theophylline, theobromine), chlorogenic acids (CGA), trigonelline, lipids, organic acids, sugars such as sucrose, free amino acids, water (moisture), fibers and minerals. These compounds undergo several physicochemical modifications due to the heat transfer. In fact, numerous chemical reactions occur, for example: simple thermal degradation, caramelisation, Maillard reaction and Strecker degradation. In our mathematical model, we consider the following key substances involved in the coffee roasting process: caffeine, trigonelline, chlorogenic acids, some organic acids, lipids, free amino acids and, among the carbohydrates, sucrose, glucose and fructose. We remark that the chemical analyses were carried out at the end of the roasting process on the substances described in the previous section, and the corresponding laboratory measurements are used in the calibration. On the other hand, sucrose, glucose, fructose and free amino acids, whose chemical analyses are not available in this study and have been parameterised from literature ranges, have been introduced for a better understanding and explanation of the chemical reactions that occur during roasting. Last but not least, this choice also provides completeness and significance to the mathematical model proposed in the sequel.

### Caffeine

Caffeine is a non-volatile compound, and it is a precursor of coffee cup quality. It mainly contributes to bitterness and astringency rather than sourness^55^. Moreover, caffeine is one of the most active methylxanthines, since it affects the cardiovascular system, the central nervous system, the gastrointestinal system and the respiratory one. It is the compound with the major consequences in the psychological sphere, improving concentration and cerebral activity^13^. Caffeine is an alkaloid, and it is relatively thermostable. This means that it is not significantly degraded during roasting. Chemical experiments have shown that slight losses may occur during the first crack of roasting, accompanied by the release of roasting gases; additionally, a small amount may be lost during the sublimation process^19^. Several studies suggest that losses of this compound may increase as the roasting temperature rises. However, caffeine is not supposed to be involved in any other reaction as a reactant; instead, it undergoes simple thermal degradation.

### Trigonelline

Trigonelline is a plant alkaloid, formed from the enzymatic methylation of nicotinic acid. It supplies the bitterness of the final beverage. In vitro studies show potential health effects, including an inhibition of the invasiveness of cancer cells^32^. Moreover, some research suggests that it can improve memory^61^. During the roasting, it is subject to a consistent degradation, as it is a thermolabile compound. It is a precursor for the formation of different classes of volatile compounds during roasting, such as pyridines and pyrroles, mainly responsible for the coffee aroma. Trigonelline, during the demethylation process, produces a B-complex vitamin, also known as niacin^1^.

### Chlorogenic acids

Chlorogenic acids comprise a diverse range of phenolic compounds, derived from the esterification of trans-cinnamic acids, specifically caffeic, ferulic, and p-coumaric, with quinic acid^52^. The most relevant CGAs are 3-CGA, 5-CGA, and 3.5-diCGA. Chlorogenic acids contribute to the acidity, astringency and bitterness of the coffee brew^19^. In recent years, numerous epidemiological and clinical studies have focused on the health benefits of coffee consumption. In particular, a lower risk of developing type 2 diabetes^38^, Parkinson’s disease^51^ and Alzheimer’s disease^17^ has been reported. In vitro and animal studies are the primary evidence supporting the notion that the beneficial properties listed above are attributed to the presence of antioxidants and other mechanisms involving chlorogenic acids^48^. CGAs are thermolabile compounds; in fact, they undergo many changes during roasting, for example, isomerisation, epimerisation and so on. These substances degrade into lower molecular compounds, including lactones and phenolic derivatives such as caffeic acid^47^. Moreover, it contributes to the formation of a phenolic acid, i.e., the ferulic acid, in the initial phases of roasting. Regarding this one, it is known that it is a precursor for some important aroma compounds, such as 4-vinylguaiacol, suggesting a degradation of ferulic acid as the roasting goes on. From a kinetic modelling perspective, the degradation of chlorogenic acids during roasting involves multiple concurrent pathways, including: hydrolysis to caffeic and quinic acids, formation of chlorogenic acid lactones and subsequent rearrangements and decarboxylation reactions. In the present model, these complex transformations are represented through a lumped kinetic pathway linking the overall CGA pool (3-CGA, 5-CGA and 3,5-diCGA) to ferulic acid as a measurable secondary phenolic product. This simplification reflects the net effect of CGA degradation observed experimentally and allows the incorporation of phenolic acid evolution within a reduced reaction network suitable for parameter estimation.

### Organic acids

Organic acids in coffee are an important source of sourness. At least thirty-eight compounds belong to this category. Individual acids have different inherent sensory properties; in fact, although citric, acetic, formic, malic, quinic, pyruvic, succinic, fumaric, tartaric, and lactic acids are all sour-tasting acids, each of them improves the aroma quality^65^. For example, the characteristic vinegar aroma of acetic acid, the burnt caramel flavour in pyruvic acid, or the pungent and fermented aroma in formic acid. Along with the sour taste, many acids such as formic, quinic, succinic and caffeic also have a perceptible bitter taste^20^. Moreover, organic acids can also help the sensorial rate by acting as savour enhancers. This is a property of fumaric, tartaric and oxalic acid. Regarding the roasting, we focus on citric, acetic, and tartaric acids, which are available through chemical analyses of roasted coffee. It is known that citric acid, already present in green coffee, can serve as a precursor to other acid breakdown products^10^. Moreover, the concentration of acetic acid tends to increase during roasting as it is a product of the Maillard reaction. The tartaric acid undergoes a simple thermal degradation during roasting.

### Lipids

Lipids are one of the main constituents of coffee, most of which are contained in the endosperm^19^. The principal constituents are triglycerides, diterpenes, triterpens and sterols. Lipids influence cream’s stability and mouthfeel. Moreover, some research suggests that they affect the stability of the foam in the beverage and are significant for flavour retention. Additionally, lipids are known to retain volatile chemical compounds within the foam layer^47^. During the roasting process, the lipid fraction of triglycerides and sterols is quite thermostable. On the other hand, among the diterpenes, we find that cafestol and kahweol can isomerise and are involved in the formation of aromatic compounds via Strecker degradation. There is scientific evidence confirming the contribution of lipids to the formation of aldehydes and pyrazines together with amino acids^36^.

### Carbohydrates and sugars

Carbohydrates make up more than 50% of the dry weight of green coffee beans. One of the most significant is sucrose, as it strongly influences the flavour and overall quality of coffee. Carbohydrates are precursors for the Maillard reaction and caramelisation, which are essential for colour and aroma development^19^. At temperatures of $140-160^\circ$C, sucrose begins to break down into simpler molecules, such as glucose and fructose. These two monosaccharides are involved in the Maillard reaction as reducing sugars. As the temperature rises above $170^\circ$C, glucose and fructose undergo caramelisation, a thermal degradation process occurring in the absence of amino compounds. At the end of the coffee roasting process, sucrose, glucose, and fructose are present in negligible quantities.

In this study, we also include the three main sugars in coffee beans (sucrose, fructose, glucose), which are not among the species we directly detect through laboratory analysis. In line with the literature, monosaccharides are present at very low levels in a cup of coffee, and the perceived sweetness is limited and influenced by sensory interactions rather than free sugars^8,40^. Therefore, we do not present the concentration profiles obtained for sucrose, fructose and glucose below; according to the literature, the numerical results show that only traces of these species remain at the end of the roasting process.

### Free amino acids

Free amino acids represent approximately the 0.5−1.0% of the total weight of the coffee bean^19^. They are essential for colour, aroma and antioxidant activity of the final coffee drink. In fact, proteins, peptides, and free amino acids, such as asparagine, alanine, and proline, are relevant for the savour of coffee, as they are needed for Maillard reactions. They act as precursors with the reducing sugars (glucose, fructose) for the formation of a wide range of volatile and non-volatile products. For instance, the formed compounds include furans, pyridines, pyrazines, pyrroles, aldehydes, and melanoidins. The latter are responsible not only for the colour of coffee but also for its antioxidant activity^35^.

### Other substances

In addition to the compounds discussed previously, other constituents of green coffee and their transformation during roasting are important to the depth of the beverage. First of all, water and minerals have to be mentioned as other major constituents of green coffee.

Moisture (i.e., water) typically represents 10−12% in green beans, and it evaporates rapidly during roasting. It influences the heat transfer and internal pressure of the bean and hence the occurrence of the first crack. Among minerals, potassium is the most present, approximately 40% of the mineral content. The remaining mineral consists of approximately 30 different elements, including sodium, magnesium, calcium and sulfur. Roasting does not particularly affect the concentration of minerals^19^.

It is well known that roasting triggers the formation of over 800 volatile molecules generally produced through Maillard, Strecker and caramelisation reactions. As previously mentioned, we can encounter furans, pyridines, pyrazines, pyrroles, aldehydes, and melanoidins. It is worth mentioning also 2-furfurylthiol^28^, methional, acetylmethylcarbinol, and guaiacol, all of which define the roasted and caramelised notes of coffee.

## Kinetic modelling

Chemical kinetics studies the rate of chemical reactions by analysing the intrinsic mechanisms in order to determine the rate of such reactions. First of all, it is essential to acknowledge that a chemical substance and its concentration are two distinct entities and must be expressed using different symbols. The recommendations of the International Union of Biochemistry and Molecular Biology^11^ are to use square brackets to denote the concentrations. For example, [*A*] is the concentration of substance *A*, [*B*] is the concentration of substance *B*, and so on. Moreover, it is useful to examine how chemical reactions can be classified. One way is according to their molecularity, which defines the number of molecules involved in a reaction. We denote an unimolecular reaction as

$$A\longrightarrow B$$

where *A* is the precursor and *B* the product. Meanwhile, a bimolecular reaction can be written as

$$A + B\longrightarrow C$$

where *A* and *B* are precursors of the product *C*.

In the proposed mathematical model, unimolecular and bimolecular reactions are assumed to follow first-order and second-order reactions, respectively. In the following, we analyse these reactions from a mathematical point of view.

In a first-order reaction, the rate is proportional to a single concentration, while in a second-order reaction it is proportional to the product of two concentrations, and so forth.

By recalling that concentrations are functions of time, in particular $[A] = [A](t)$, we simplify the notation, by using [*A*] instead of [*A*](*t*). With this assumption, the rate *r* of a first-order reaction A $\longrightarrow$ B can be expressed as

1 $$\begin{aligned} r = \dfrac{d [B]}{dt} = - \dfrac{d [A]}{dt} = k [A] = k ([A]_0 - [B]), \end{aligned}$$

where $[A]_0$ is the initial concentration of *A*, zero is the initial concentration of *B*, and *k* is a first-order rate constant. Note that (1) can be equivalently rewritten as:

2 $$\begin{aligned} \begin{aligned} \dfrac{d[A]}{dt}&= - k[A],\\ \dfrac{d[B]}{dt}&= k[A]. \end{aligned} \end{aligned}$$

Mathematically speaking, there is no difference in whether the rate is defined in terms of the formation of products or the degradation of reactants. Note that, from (1), the rate *r* provides two alternative characterisations. With the hypothesis used above, that $[A] = [A]_0$ and $[B] = 0$ at time $t = 0$, from (2) we have that [*A*] and [*B*] are related by

$$\begin{aligned} [A] + [B] = [A]_0. \end{aligned}$$

By standard arguments of mathematical analysis^12^, we have that the solution of (1) is given by

3 $$\begin{aligned} [B] = [A]_0(1 - e^{-kt}). \end{aligned}$$

The reaction A + B $\longrightarrow$ C can be described by

4 $$\begin{aligned} \begin{aligned} \dfrac{d[A]}{dt}&= - k[A][B],\\ \dfrac{d[B]}{dt}&= - k[A][B],\\ \dfrac{d[C]}{dt}&= k[A][B]. \end{aligned} \end{aligned}$$

If we assume that the initial concentrations of *C* is zero, we can write:

5 $$\begin{aligned} r = \dfrac{d[C]}{dt} = k[A][B] = k([A]_0 -[C])([B]_0 -[C]), \end{aligned}$$

where now *k* is a second-order rate constant, $[A]_0$ and $[B]_0$ are initial concentrations of *A* and *B*, respectively. In fact, the system of Ordinary Differential Equations (ODEs) (4) gives

$$\begin{aligned} \begin{aligned} \dfrac{d([A]+[C])}{dt}&= 0,\\ \dfrac{d([B] + [C])}{dt}&= 0. \end{aligned} \end{aligned}$$

Then, with the above assumption that $[C] = 0$ at time $t = 0$, we obtain

$$\begin{aligned} [A]+[C]=[A]_0,\qquad [B]+[C]=[B]_0 \end{aligned}$$

which justifies the last equality in (5). Moreover, under the assumption that $[A]_0 \ne [B]_0$, by rearranging (5) as

$$\begin{aligned} \dfrac{d[C]}{([A]_0 -[C])([B]_0 -[C])} = k\,dt, \end{aligned}$$

a simple integration yields

$$\begin{aligned} \ln \left( \dfrac{[A]_0([B]_0 - C)}{[B]_0([A]_0 - C)}\right) = k([B]_0 - [A]_0)t. \end{aligned}$$

Finally, the solution for (5) is

6 $$\begin{aligned} [C] = \dfrac{[A]_0 [B]_0(e^{k([B]_0 -[A]_0)t}-1)}{[B]_0e^{k([B]_0 -[A]_0)t}-[A]_0}. \end{aligned}$$

It is useful to notice that this expression has denominator different from zero so its qualitative behaviour resembles the one of expression in (3); moreover, if concentrations $[\cdot ]$ are expressed as percentage mass fractions on a dry-weight basis ($\%\,w/w$) and time is measured in seconds, then first-order rate constants have units $\mathrm{s}^{-1}$, whereas second-order rate constants have units $(\%\,w/w)^{-1}\,\mathrm{s}^{-1}$.

In many cases, a more complex kinetics can occur in chemical reactions. The previously discussed chemical reactions are among the irreversible ones. In principle, chemical reactions can be reversible, i.e., reactants form products, while the products can simultaneously revert to form reactants, and the reaction can be represented as

$$A\underset{k_2}{\overset{k_1}{\rightleftharpoons}}B$$

In this circumstance, if we assume that the initial concentration of *B* is zero, the rate *r* is given by

7 $$\begin{aligned} r = \dfrac{d [B]}{dt} = k_1([A]_0 - [B]) - k_2[B] = k_1[A]_0 - (k_1 + k_2)[B], \end{aligned}$$

and the corresponding ODEs are

8 $$\begin{aligned} \begin{aligned} \dfrac{d [A]}{dt}&= - k_1[A] + k_2[B],\\ \dfrac{d [B]}{dt}&= k_1[A] - k_2[B]. \end{aligned} \end{aligned}$$

In detail, under the assumption $[B]_0 = 0$ and from (8) we have that $[A] + [B] = [A]_0$ and, a solution for (7) is given by

9 $$\begin{aligned} [B] = \dfrac{[A]_0 k_1}{k_1 + k_2}\left( 1 - e^{-(k_1 + k_2) t}\right) = [B]_{\infty }\left( 1 - e^{-(k_1 + k_2) t}\right) , \end{aligned}$$

where $[B]_{\infty }$ is the concentration of the species *B* as $t \rightarrow +\infty$.

In classical chemical kinetics, rate constants are derived experimentally by analysing the temporal evolution of concentration, assuming that the reaction is of first-order^64^. From (9) we have

$$\begin{aligned} [B]_{\infty } - [B] = [B]_{\infty }e^{-(k_1 + k_2) t}, \end{aligned}$$

then,

$$\begin{aligned} \ln {([B]_{\infty } - [B])} = \ln {[B]_{\infty }} -(k_1 + k_2) t. \end{aligned}$$

Hence, a plot of $\ln {([B]_{\infty } - [B])}$ against *t* gives a straight line with slope $-(k_1 + k_2)$, which can be used to extract information on the rate constants. However, this requires knowledge of the asymptotic concentration $[B]_{\infty }$, which, in general, can be imprecise or difficult to obtain. For this reason, in most cases, the so-called Guggenheim method^31^ is preferable, since it provides an estimate of the rate constants by neglecting the dependence on $[B]_{\infty }$. In our work, we aim to calibrate the rate constants using a constrained optimisation procedure based on the available experimental data.

We conclude this section with a discussion of the influence of temperature on rate constants. The modern theory describing the impact of temperature on rate constants is based on the work of chemists *van’t Hoff* and *Arrhenius*^41^. Their contribution provides an exact relationship linking kinetic observations with the known properties of equilibrium constants.

We consider a reversible reaction, whose dynamics is described by Equation (7), and we suppose that the reaction is in chemical equilibrium, so that an equilibrium constant *K* can be defined as the ratio of product concentration to reactant concentration. The dependence of *K* on temperature *T* is described by the van’t Hoff equation, i.e.,

10 $$\begin{aligned} \dfrac{d \ln {K}}{dT} = \dfrac{\Delta H^0}{RT^2}, \end{aligned}$$

where *R* is the universal gas constant and $\Delta H^0$ is the reaction’s enthalpy variation. From the chemical kinetic laws, under equilibrium conditions^33^, we have that

$$\begin{aligned} K = \frac{k_1}{k_2}, \end{aligned}$$

where $k_1$ and $k_2$ are the rate constants of the forward and reverse reaction, respectively. Hence, we can rearrange (10) as

11 $$\begin{aligned} \dfrac{d \ln {\left( \tfrac{k_1}{k_2}\right) }}{dT} = \dfrac{d \ln {k_1}}{dT} - \dfrac{d\ln {k_2}}{dT} = \dfrac{\Delta H^0}{RT^2}. \end{aligned}$$

Hence, we obtain an expression for the rate constants in terms of the difference in their variations. We split equation (11) in such a way to obtain two separate evolutions for each rate constant $k_1$ and $k_2$, i.e.,

12 $$\begin{aligned} \begin{aligned} \dfrac{d\ln {k_1}}{dT}&= \dfrac{\Delta H^0_1}{RT^2} + \lambda , \\ \dfrac{d\ln {k_2}}{dT}&= \dfrac{\Delta H^0_2}{RT^2} + \lambda , \end{aligned} \end{aligned}$$

where $\lambda$ is an integration constant. Arrhenius, supplied by experimental data, postulates that the constant $\lambda$ is zero and that the temperature dependence of any rate constant *k* is described by the following differential equation

13 $$\begin{aligned} \dfrac{d\ln {k}}{dT} = \dfrac{E_a}{RT^2}, \end{aligned}$$

where $E_a$ is the so-called activation energy, i.e., a kinetic parameter representing the minimum energy required to trigger a chemical reaction. A simple integration with respect to *T*, and by exponentiation, yields an expression for the rate constant *k* in terms of the temperature *T*, i.e.,

14 $$\begin{aligned} \displaystyle k(T) = \alpha e^{-\dfrac{E_a}{RT}}, \end{aligned}$$

where $\alpha$ is the constant of integration. Formula (14) is called the Arrhenius law.

## The mathematical model

We translate the chemical concepts presented in the previous section into mathematical language, making use of the tools described above. We take into account the chemical compounds identified in Sections “Experimental analyses” and "Behaviour of chemical compounds during roasting and modelling assumptions", i.e., caffeine ($\textrm{CF}$), tartaric acid ($\textrm{TA}$), acetic acid ($\textrm{AA}$), citric acid ($\textrm{CA}$), trigonelline ($\textrm{TR}$), chlorogenic acids ($\textrm{CGA}$), ferulic acid ($\textrm{FA}$), lipids ($\textrm{LP}$), sucrose ($\textrm{SUC}$), glucose ($\textrm{GLC}$), fructose ($\textrm{FRU}$), and free amino acids ($\textrm{FAM}$).

We know that, during roasting, sucrose (being a disaccharide) is subject to thermal degradation, and in particular, it acts as a precursor for the monosaccharides considered in this study, i.e., glucose and fructose; this represents a first-order degradation step. Moreover, it is well known that, during roasting, chlorogenic acids act as precursors of several phenolic degradation products. In the proposed model, the transformation of $\textrm{CGA}$ into ferulic acid is represented as a lumped unimolecular pathway, accounting for the net formation of ferulic acid from the $\textrm{CGA}$ pool through multiple intermediate reactions. This modelling choice is motivated by the availability of experimental data for ferulic acid and by the need to limit model complexity while preserving chemical plausibility. Since we are only tracking a fixed number of substances, and the remaining ones available are not directly related as precursor-product pairs in specific chemical reactions, it is difficult to construct a chemical scheme that connects all the listed compounds. For this reason, and to highlight the presence of the most important chemical reactions in the coffee roasting process, we introduce a “sink” variable that accounts for other substances ($\textrm{OTH}$) that are not directly quantified by our chemical analyses. In fact, the Maillard reaction occurs between reducing sugars (glucose and fructose) and amino acids. The products of this reaction, which include both volatiles and non-volatile compounds (e.g., melanoidines, pyridines, and so on), are collected within $\textrm{OTH},$ and the reaction is modelled by a second-order kinetics, as we have two reactants combining to form products.

In the context of the Maillard reaction, acetic acid can also be considered a Maillard product involving reducing sugars ($\textrm{GLC}$, $\textrm{FRU}$), hence in our formulation $\textrm{AA}$ is treated as an explicit product of second-order kinetics in which $\textrm{FAM}$ acts as the second precursor. Another important reaction in which sugars are the only precursors is caramelisation, whose products are stored in $\textrm{OTH}$; accordingly, we model a first-order loss of each monosaccharide.

**Fig. 1:**
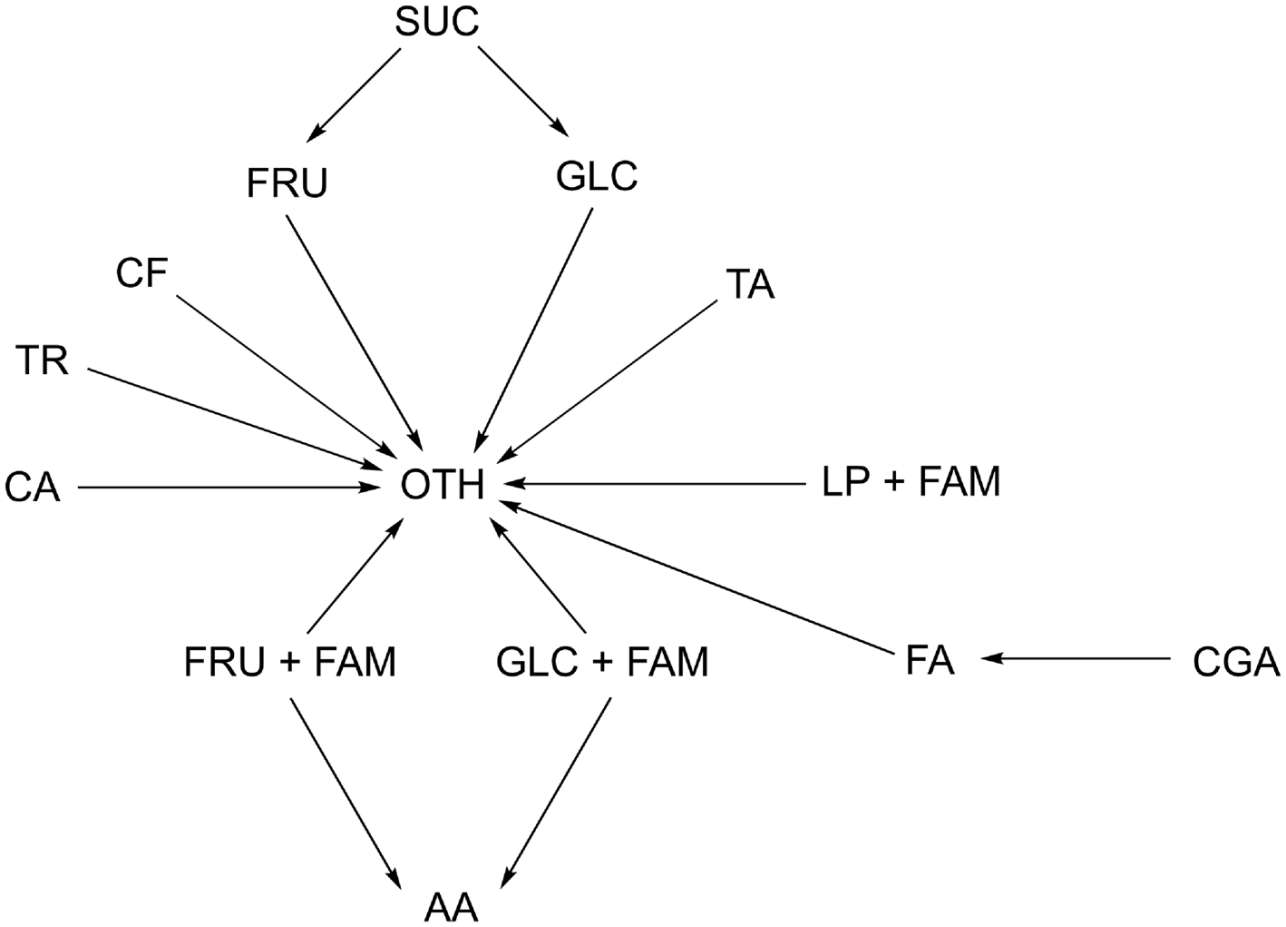
Chemical reactions scheme of the proposed coffee-roasting kinetic model. State variables are: $\textrm{CF}$ (caffeine), $\textrm{TA}$ (tartaric acid), $\textrm{AA}$ (acetic acid), $\textrm{CA}$ (citric acid), $\textrm{TR}$ (trigonelline), $\textrm{CGA}$ (chlorogenic acids), $\textrm{FA}$ (ferulic acid), $\textrm{LP}$ (lipids), $\textrm{SUC}$ (sucrose), $\textrm{FRU}$ (fructose), $\textrm{GLC}$ (glucose), $\textrm{FAM}$ (free amino acids), and $\textrm{OTH}$ (other substances). Arrows indicate precursor–product relationships.

A further reaction that can be taken into account in our model is Strecker degradation, which involves lipids (in particular kahweol and cafestol) and amino acids ($\textrm{FAM}$) as precursors, whose products are mainly aldehydes and pyrazines. Thus, we have a second-order reaction in which $\textrm{LP}$ and $\textrm{FAM}$ act as precursors and, again, $\textrm{OTH}$ is the product. Trigonelline is thermally labile and generates products, such as niacin, that are not chemically analysed. Therefore, its consumption is represented as a first-order kinetic loss into $\textrm{OTH}$. The same discussion can be extended to citric and ferulic acid. Finally, for caffeine and tartaric acid, the chemical discussion in Section "Behaviour of chemical compounds during roasting and modelling assumptions" suggests that they are not considered precursors of other specific measured compounds and primarily undergo simple thermal degradation during roasting. However, to keep the reaction network connected and to impose mass conservation in our system, we route their first-order consumption into $\textrm{OTH}$. With this convention, the chemical network remains connected, and the total mass is conserved, i.e., the sum of the time derivatives over all concentration variables is zero by construction. Figure 1 provides a schematic representation of the reaction network adopted in this work. Arrows indicate precursor-product relationships.

Let $I = [0, \tau ]$ be the roasting time interval expressed in seconds. For $t \in I$, the mathematical roasting model can be formulated as follows

15 $$\begin{aligned} & \dfrac{d[\textrm{CF}]}{dt} = -k_1 [\textrm{CF}] , \end{aligned}$$

16 $$\begin{aligned} & \dfrac{d[\textrm{TA}]}{dt} = -k_2 [\textrm{TA}], \end{aligned}$$

17 $$\begin{aligned} & \dfrac{d[\textrm{SUC}]}{dt} = -(k_3+k_4) [\textrm{SUC}], \end{aligned}$$

18 $$\begin{aligned} & \dfrac{d[\textrm{FRU}]}{dt} = k_3 [\textrm{SUC}] - k_5[\textrm{FRU}][\textrm{FAM}] - k_6[\textrm{FRU}] - k_7[\textrm{FRU}][\textrm{FAM}], \end{aligned}$$

19 $$\begin{aligned} & \dfrac{d[\textrm{GLC}]}{dt} = k_4 [\textrm{SUC}] - k_8 [\textrm{GLC}][\textrm{FAM}] - k_9[\textrm{GLC}] - k_{10}[\textrm{GLC}][\textrm{FAM}], \end{aligned}$$

20 $$\begin{aligned} & \dfrac{d[\textrm{AA}]}{dt} = 2k_{7} [\textrm{FRU}][\textrm{FAM}] + 2k_{10} [\textrm{GLC}][\textrm{FAM}], \end{aligned}$$

21 $$\begin{aligned} & \dfrac{d[\textrm{CA}]}{dt} = -k_{11} [\textrm{CA}], \end{aligned}$$

22 $$\begin{aligned} & \dfrac{d[\textrm{TR}]}{dt} = -k_{12} [\textrm{TR}], \end{aligned}$$

23 $$\begin{aligned} & \dfrac{d[\textrm{CGA}]}{dt} = -k_{13} [\textrm{CGA}], \end{aligned}$$

24 $$\begin{aligned} & \dfrac{d[\textrm{FA}]}{dt} = k_{13} [\textrm{CGA}] - k_{14} [\textrm{FA}], \end{aligned}$$

25 $$\begin{aligned} & \dfrac{d[\textrm{LP}]}{dt} = -k_{15} [\textrm{LP}][\textrm{FAM}], \end{aligned}$$

26 $$\begin{aligned} & \dfrac{d[\textrm{FAM}]}{dt} = -k_5[\textrm{FRU}][\textrm{FAM}] - k_7[\textrm{FRU}][\textrm{FAM}] - k_8 [\textrm{GLC}][\textrm{FAM}] \nonumber \\ & \qquad \quad \quad \,\,\,\,\, -k_{10}[\textrm{GLC}][\textrm{FAM}] - k_{15}[\textrm{LP}][\textrm{FAM}], \end{aligned}$$

27 $$\begin{aligned} & \dfrac{d[\textrm{OTH}]}{dt} = k_1[\textrm{CF}] + k_2 [\textrm{TA}] + 2k_5[\textrm{FRU}][\textrm{FAM}] +k_6[\textrm{FRU}] \nonumber \\ & \qquad \quad \quad \,\,\,\,\, +2k_8[\textrm{GLC}][\textrm{FAM}] + k_9[\textrm{GLC}] +k_{11} [\textrm{CA}] + k_{12} [\textrm{TR}] \nonumber \\ & \qquad \quad \quad \,\,\,\,\, +k_{14} [\textrm{FA}] + 2k_{15} [\textrm{LP}][\textrm{FAM}], \end{aligned}$$

where $k_i$, $i = 1,2, \dots , M$, $M=15$, are the reaction rates, and, for the sake of simplicity, we omit the dependence on *t*. Note that the ODE system (15) - (27) must be equipped with appropriate initial conditions, and the factors 2 appearing as a multiplicative constant in some of the Equations (15) - (27) are introduced solely for mass-balance. In fact, we observe that the total mass is conserved by the proposed ODE system for the evolution of chemical compounds during the coffee roasting process. In particular, if we define the total mass at time *t* (in dry-weight basis) as

$$\begin{aligned} \begin{aligned} M_{tot}(t) =&[\textrm{CF}]+[\textrm{TA}]+[\textrm{AA}]+[\textrm{CA}]+[\textrm{TR}]+[\textrm{CGA}]+[\textrm{FA}]+\\&+[\textrm{LP}]+ [\textrm{SUC}]+[\textrm{FRU}]+[\textrm{GLC}]+[\textrm{FAM}]+[\textrm{OTH}], \end{aligned} \end{aligned}$$

then, a direct summation of Equations (15)–(27) yields

28 $$\begin{aligned} \dfrac{dM_{tot}}{dt} = 0. \end{aligned}$$

Equation (28) implies that $M_{tot}(t) = M_{tot}(0)$ $\forall t \in I$. In particular, the initial conditions are normalised such that $M_{tot}(0) = 100$, and then $M_{tot}(t) = 100$ $\forall t \in I$.

Let $T_b: I \rightarrow (0, \infty )$ be the roasting temperature profile of the coffee beans. For $i = 1,2, \dots , M,$ we assume that the reaction rates describing the evolution of the concentrations of the considered substances follow the Arrhenius law, as stated in formula (14), i.e.,

29 $$\begin{aligned} k_i = k_i(T_b(t)) = \alpha _{i} e^{-\dfrac{E_{a,i}}{R\,T_b(t)}}, \end{aligned}$$

where $\alpha _i> 0$, $E_{a,i} \ge 0$ are suitable kinetic parameters.

### The calibration procedure

We now describe the calibration problem associated with the mathematical model of coffee roasting (15) - (27); the goal is to estimate the set of kinetic parameters, $\alpha _i$ and $E_{a,i}$, $i = 1,2,\dots ,M,$ capable of reproducing the experimentally measured composition of roasted coffee at the end of the process, i.e. at time $t = \tau$.

First of all, let $\mathbb {R}_{\ge 0}$ be the set of non-negative real numbers and let $N = 8$ be the number of measured aforementioned species, the first eight in (30).

We define ${\textbf{c}}(t)\in \mathbb {R}_{\ge 0}^{N + 5}$ the column vector of the concentration values, whose unit of measurement is expressed as a percentage on a dry matter basis, and whose entries are ordered in the following way

30 $$\begin{aligned} \begin{aligned}&[\textrm{CF}],\,[\textrm{TA}],\,[\textrm{AA}],\,[\textrm{CA}],\,[\textrm{TR}],\, [\textrm{CGA}],\,[\textrm{FA}],\,[\textrm{LP}],\\&[\textrm{SUC}],\,[\textrm{FRU}],\, [\textrm{GLC}],\,[\textrm{FAM}],\,[\textrm{OTH}], \end{aligned} \end{aligned}$$

Given temperature profile $T_b(t)$ and the vector

$$\begin{aligned} \boldsymbol{\theta }= (\alpha _1,\, \alpha _2,\, \dots ,\, \alpha _M,\, E_{a,1},\, E_{a,2},\, \dots ,\, E_{a,M})^T \in \mathbb {R}^{2M}, \end{aligned}$$

whose entries are the parameters of Arrhenius law, the system of ODEs (15) - (27) can be rewritten in a more compact form as

31 $$\begin{aligned} \frac{d{\textbf{c}}}{dt} = {\textbf{f}}\big ({\textbf{c}}(t),T_b(t);\boldsymbol{\theta }\big ), \qquad {\textbf{c}}(0) = {\textbf{c}}_0, \end{aligned}$$

where ${\textbf{f}}$ is specified component-wise by (15) - (27) and ${\textbf{c}}_0=(c_{1,0},c_{2,0},\dots , c_{N+5,0})^T\in \mathbb {R}_{\ge 0}^{N+5}$ denotes the vector of initial concentrations determined as follows. Let *m* be the weight of the green coffee bean, and $m_j$, $j=1,2,\dots , N+4,$ be the weight of the species *j* in the green coffee bean, with the ordering given in (30), then we have

$$\begin{aligned} m_{N+5}=m-\sum _{j=1}^{N+4}m_j.\end{aligned}$$

The initial conditions are

$$\begin{aligned}{c}_{j,0}=\frac{m_j}{m}\cdot 100,\qquad j=1,2,\dots ,N+5,\end{aligned}$$

where for $j=1,2,\dots ,N+4,$ $m_j$ are obtained as the mean value of the range given in the literature, see Table 1, as we have only the final laboratory measurements.

Under these assumptions, for each admissible $\boldsymbol{\theta }$, the initial value problem (31) has a unique solution ${\textbf{c}}(t)$ defined on the roasting interval *I*. Note that the solution ${\textbf{c}}(t)$ depends on $\boldsymbol{\theta }$. Given $\boldsymbol{\theta }$, by using the above-described model, we can compute the numerical concentrations at the final roasting time, the vector ${\textbf{c}}(\tau )$. It depends on $\boldsymbol{\theta }$, and we can compare its first *N* entries with the experimental data obtained from the chemical analyses of the roasted beans, denoted by ${\bar{c}}_j(\tau )$, $j=1,2,\dots ,N$. In particular, this allows us to calibrate the model, that is, choose $\boldsymbol{\theta }$. Hence the calibration procedure of the mathematical coffee roasting model is performed using the final concentrations of the measured species. Since these species can span different orders of magnitude, instead of minimising the simple sum of squares of the relative errors at the final time, we define a scale-invariant objective function based on the logarithmic residuals, defined as follows

32 $$\begin{aligned} r_j(\boldsymbol{\theta }) = \log \left( \frac{c_j(\tau )}{{\bar{c}}_j(\tau )}\right) , \quad j=1,2,\dots ,N. \end{aligned}$$

Note that under the assumptions made in the considered kinetic model, all concentrations are strictly positive, so that (32) is well-defined. Moreover, if

$$\begin{aligned} c_j(\tau ) = {\bar{c}}_j(\tau )(1+\delta ), \end{aligned}$$

then, when $c_j(\tau )\approx {\bar{c}}_j(\tau )$, $|\delta | \ll 1$ and (32) can be written as

$$\begin{aligned} r_j(\boldsymbol{\theta }) = \log (1+\delta ) \approx \delta = \frac{c_j(\tau )-{\bar{c}}_j(\tau )}{{\bar{c}}_j(\tau )}, \end{aligned}$$

i.e., $|r_j|$ approximates the usual relative error of the species *j*.

By collecting all the log-residuals into the vector ${\textbf{r}}(\boldsymbol{\theta })$, i.e.,

$$\begin{aligned} {\textbf{r}}(\boldsymbol{\theta }) = \big (r_1(\boldsymbol{\theta }), r_2(\boldsymbol{\theta }), \dots , r_N(\boldsymbol{\theta })\big )^T \in \mathbb {R}^{N}, \end{aligned}$$

we can consider the following objective function

33 $$\begin{aligned} F(\boldsymbol{\theta }) = \Vert {\textbf{r}}(\boldsymbol{\theta })\Vert _2^2 = \sum _{j}^{N} r_j(\boldsymbol{\theta })^2. \end{aligned}$$

This dimensionless function measures the overall discrepancy between the simulated and experimental final compositions.

The kinetic parameters that we want to estimate, i.e., the entries of the vector $\boldsymbol{\theta }\in \mathbb {R}^{2M}$, are restricted to the following admissible set

34 $$\begin{aligned} \Theta _{\text {ad}} = \Big \{\boldsymbol{\theta }\in \mathbb {R}^{2M} : 0 < \alpha _i \le \alpha _i^{\max },\; E_{a,i}^{\min } \le E_{a,i} \le E_{a,i}^{\max }, \; i=1,\dots ,M\Big \}, \end{aligned}$$

where the bounds are introduced to enforce positivity and exclude unphysical values.

For any $\boldsymbol{\theta }\in \Theta _{\textrm{ad}}$, a solution ${\textbf{c}}(t)$ must satisfy the kinetic model, i.e., the ODE system (31), with initial conditions determined by the considered green coffee, which are literature-based, at this stage. Thus, the calibration procedure, i.e., the parameter estimation problem, can be written as the following nonlinear constrained optimisation problem

35 $$\begin{aligned} \min _{\boldsymbol{\theta }\in \Theta _{\textrm{ad}}} F(\boldsymbol{\theta }). \end{aligned}$$

We note that the objective function is based on $N=8$ measurements obtained at the end of the roasting process, while the unknown kinetic parameters are $2M=30$, so the problem is underdetermined. Hence, we expect that the parameters vector $\boldsymbol{\theta }$ is not uniquely identifiable. Moreover, despite the underdetermined nature of the problem and the initial conditions taken from the literature, the following numerical results confirm the flexibility of the proposed method.

### Numerical implementation

The optimisation problem (35) is solved numerically by combining a variable-step, variable-order ODE solver, based on the numerical differentiation formulas of orders 1 to 5 used to obtain an approximate solution of the initial value problem (31) and a constrained non-linear least-squares algorithm. Moreover, since these kinds of problems are usually non-convex, the objective function may have many minima. For this reason, we adopt a multistart strategy to mitigate this issue.

First of all, for the numerical solution, it is convenient to reparametrise the Arrhenius law in terms of the reference rate constants, $k_{i}^{\text {ref}}$. Given a reference temperature $T_{\text {ref}}$, we can write

36 $$\begin{aligned} k_i(T)= k_{i}^{\text {ref}}\exp \left[ -\frac{E_{a,i}}{R}\left( \frac{1}{T} - \frac{1}{T_{\textrm{ref}}}\right) \right] , \quad i = 1, 2, \dots , M. \end{aligned}$$

Observe that $k_{i}^{\text {ref}} = k_i(T_{\text {ref}})> 0$ is the rate constant at temperature $T_{\text {ref}}$, whereas $E_{a,i} \ge 0$ is the activation energy. This formulation is algebraically equivalent to the standard Arrhenius law (29) with

37 $$\begin{aligned} \alpha _i = k_{i}^{\text {ref}}e^{\frac{E_{a,i}}{R T_{\textrm{ref}}}}, \quad i = 1,2,\dots ,M. \end{aligned}$$

As stated in Schwaab and Pinto^57^, the use of formula (36) leads to better scaling and reduces the correlation between the fitted parameters. The admissible set (34) must be therefore specified by bounds on $(k_{i}^{\text {ref}},E_{a,i})$, $i = 1,2, \dots , M$. These bounds are chosen to encode basic physicochemical knowledge on the evolution of the studied compounds during coffee roasting. In particular, for $i = 1,2, \dots , M,$

(*b*1): the activation energies $E_{a,i}$ are restricted to the interval with limits selected in accordance with typical values reported for the main chemical reactions involved, i.e. thermal degradation, the Maillard reaction, and so forth. For instance, based on van Boekel^63^, we typically select a range of the order of 40-$150~\mathrm {kJ/mol}$ for the activation energies;
  $$\begin{aligned}E_{a,i}^{\min } \le E_{a,i} \le E_{a,i}^{\max },\end{aligned}$$
(*b*2): the rate constants at temperature $T_{\text {ref}}$, i.e., the parameters $k_{i}^{\text {ref}} = k_i(T_{\text {ref}})$, are bounded in a dimensionally consistent way. First-order constants, whose unit is $s^{-1}$, are constrained as $k^{\textrm{ref}}_i\in (0,0.1]$ for $i\in \{1,2,3,4,6,9,11,12,13,14\}$, whereas second-order constants, whose unit is $(\%\,w/w)^{-1}s^{-1}$, are constrained as $k^{\textrm{ref}}_i\in (0,0.001]$ for $i\in \{5,7,8,10,15\}$.

From the choice of bounds (*b*1), (*b*2) and from Equations (36), (37) we have that

$$\begin{aligned} \boldsymbol{\theta }\in \Theta _{\text {ad}} \iff \boldsymbol{{\tilde{\theta }}} \in {\tilde{\Theta }}_{\text {ad}}, \end{aligned}$$

where

$$\begin{aligned} \boldsymbol{{\tilde{\theta }}} = (k^{\text {ref}}_1,\, k^{\text {ref}}_2,\, \dots ,\, k^{\text {ref}}_M,\, E_{a,1},\, E_{a,2},\, \dots ,\, E_{a,M})^T \in \mathbb {R}^{2M}, \end{aligned}$$

and

38 $$\begin{aligned} {{\tilde{\Theta }}}_{\text {ad}} = \Big \{\boldsymbol{{\tilde{\theta }}} \in \mathbb {R}^{2M} : 0 < k^{\text {ref}}_i \le k_i^{\max },\; E_{a,i}^{\min } \le E_{a,i} \le E_{a,i}^{\max }, \; i=1,\dots ,M\Big \}. \end{aligned}$$

For each admissible parameter vector $\boldsymbol{\theta }\in \Theta _{\text {ad}}$ (or equivalently $\tilde{\boldsymbol{\theta }} \in {\tilde{\Theta }}_{\text {ad}}$) the kinetic model (31) is integrated over the interval *I* using a stiff ODE algorithm with the initial concentration ${\textbf{c}}_0$ and the bean temperature profile $T_b(t)$ as input. Given the final measured concentrations i.e., the values ${\bar{c}}_j(\tau )$, $j=1,2,\dots , N$, we evaluate the objective function $F(\boldsymbol{\theta })$ employing the simulated final concentration obtained from the numerical solution. Consequently, the constrained non-linear least-squares problem (35) is solved.

As previously noted, these kinds of optimisation problems are usually non-convex, which means that the global minimum is strongly affected by the choice of the starting point for the non-linear minimisation routine. For this reason, we adopt a multi-start strategy to reduce this dependence on the initial guess.

We summarise all the steps required for the implementation of the calibration procedure in Algorithm 1.

**Algorithm 1:**
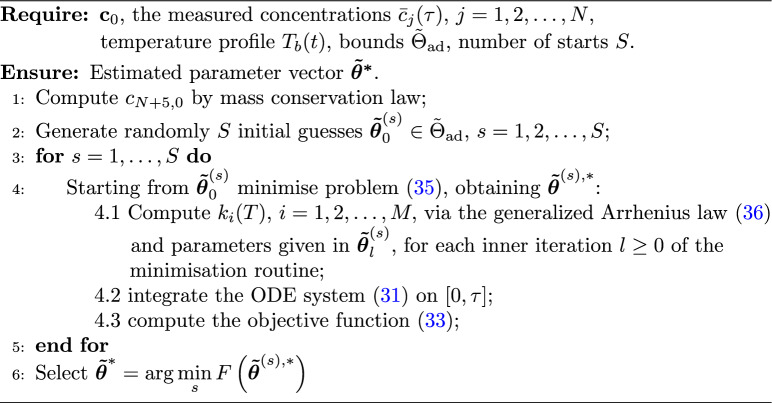
Calibration of the kinetic parameters

Note that each evaluation of the objective function *F* within the minimisation routine, as described in Algorithm 1, requires the numerical solution of the ODE system (31) with the current kinetic parameters.

Algorithm 1 has been implemented in MATLAB^44^. In particular, we use the stiff ODE solver ode15s^59^ for the numerical solution of the initial value problem (31) and the MATLAB function lsqnonlin for the non-linear constrained optimisation problem (35) with explicit lower and upper bounds. In particular, the function ode15s is equipped with the option ‘NonNegative’ that ensures non-negativity of all chemical compounds. The optimisation performed in MATLAB by lsqnonlin uses the following options:

- Algorithm = ‘levenberg-marquardt’;
- MaxIterations = 300;
- MaxFunctionEvaluations = $$5\,\times 10^4$$;
- StepTolerance = $$10^{-10}$$;
- FunctionTolerance = $$10^{-10}$$.

As discussed in the previous subsection, the calibration problem is underdetermined. Hence, the trust-region-reflective algorithm is not applicable, and lsqnonlin uses the *Levenberg-Marquardt*^23^ method. Moreover, in the presence of bound constraints, lsqnonlin modifies the *Levenberg-Marquardt* iterations as follows: if an iterative solution $\boldsymbol{{\tilde{\theta }}} \in \mathbb {R}^{2M}$ lies outside the bounds, the algorithm projects the step onto the nearest feasible point^43^. In detail, the projection operator $P:\mathbb {R}^{2M}\rightarrow {{\tilde{\Theta }}}_{\text {ad}}$, which projects infeasible points onto the feasible region, is defined as follows. Let $\boldsymbol{\ell },{\boldsymbol{u}} \in \mathbb {R}^{2M}$ be the lower and upper bounds vectors, respectively, defined as

$$\begin{aligned} \boldsymbol{\ell }=\big (0,\dots ,0,\,E_{a,1}^{\min },\dots ,E_{a,M}^{\min }\big )^{\!T},\qquad \boldsymbol{u}=\big (k_1^{\max },\dots ,k_M^{\max },\,E_{a,1}^{\max },\dots ,E_{a,M}^{\max }\big )^{\!T}. \end{aligned}$$

Given $\boldsymbol{{\tilde{\theta }}}\in \mathbb {R}^{2M}$, for $j=1,2,\dots , 2M,$ the operator *P* is given by:

$$\begin{aligned} \left( P\left( \boldsymbol{{\tilde{\theta }}}\right) \right) _j= {\left\{ \begin{array}{ll} \ell _j, & {{\tilde{\theta }}}_j<\ell _j,\\ u_j, & {{\tilde{\theta }}}_j>u_j,\\ {{\tilde{\theta }}}_j, & \text {otherwise}, \end{array}\right. } \qquad \text {i.e.,}\qquad P(\boldsymbol{{\tilde{\theta }}})=\min \big (\max (\boldsymbol{{\tilde{\theta }}},\boldsymbol{\ell }),\boldsymbol{u}\big ). \end{aligned}$$

Bound enforcement has been verified after the optimisation routine by checking that the optimal parameter $\boldsymbol{{\tilde{\theta }}}^*$ satisfies the prescribed bounds. In Tables 6 and 7, there are the optimal parameters obtained by choosing $S=30$.

## Results and discussion

In this section, we present the experimental data used for the calibration of the kinetic model.

### Green coffee composition

We first specify the initial composition of green coffee used as initial condition for numerically solving the ODE system in (31). Table 1 reports representative values for the main chemical species considered in the mathematical model for *Coffea Arabica* and *Coffea Canephora* (Robusta). These values are available among the various literature investigating the green coffee composition, see for example^19,47^ for more details.

**Table 1:** Representative literature ranges or mean chemical composition of green Arabica and Robusta coffee. Values are expressed in percentage as mass fractions on a dry-weight basis ($\%\,w/w$). For each coffee type, the fraction of other substances $\textrm{OTH}$ is defined as 100 minus the sum of the listed compounds.

Compound	Arabica ($\%\,w/w$)	Robusta ($\%\,w/w$)
Caffeine ($\textrm{CF}$)	0.8–1.4	1.7–4.0
Tartaric acid ($\textrm{TA}$)	0.02	0.02
Sucrose ($\textrm{SUC}$)	6.0–9.0	0.9–4.0
Fructose ($\textrm{FRU}$)	0.05	0.2
Glucose ($\textrm{GLC}$)	0.05	0.2
Free amino acids ($\textrm{FAM}$)	0.5	0.8–1.0
Acetic acid ($\textrm{AA}$)	0.02	0.025
Citric acid ($\textrm{CA}$)	0.2–0.6	0.2–0.7
Trigonelline ($\textrm{TR}$)	0.6–1.3	0.3–0.9
Chlorogenic acids ($\textrm{CGA}$)	4.0–8.0	7.0–10.0
Ferulic acid ($\textrm{FA}$)	0.03–0.09	0.04–0.12
Lipids ($\textrm{LP}$)	10–17	11

### Results of chemical analyses

We consider four roasted coffee powders provided by Gambilongo Caffè srl: two *Coffea arabica L.* samples (Mexico, Rwanda) and two *Coffea canephora Pierre ex A. Froehner* samples (Nicaragua, Indonesia). For each roasted sample, chemical analyses are carried out to quantify the concentration of caffeine, trigonelline, selected chlorogenic acids, ferulic acid, organic acids and lipids according to the procedure described in Section “Experimental analyses”. The corresponding final compositions are summarised in Table 2 as mean ± standard deviation over three measurements. The chemical composition of the roasted coffee powders showed marked differences between Coffea arabica (Mexico, Rwanda) and Coffea canephora (Nicaragua, Indonesia) samples. As expected, Robusta coffees exhibited higher caffeine and chlorogenic acid contents than Arabica, in agreement with previously reported species-dependent differences^19^. Caffeine levels ranged from 8491 to 8615 $\mathrm{mg} \cdot \mathrm{kg}^{-1}$ in Arabica and 15258 to 16607 $\mathrm{mg}\cdot \mathrm{kg}^{-1}$ in Robusta. Trigonelline concentrations were inversely correlated, being higher in Arabica ($\approx 310$ $\mathrm{mg} \cdot \mathrm{kg}^{-1}$) and lower in Robusta ($\approx 224$ $\mathrm{mg}\cdot \mathrm{kg}^{-1}$), consistent with the known thermolability of this compound during roasting and its species-dependent precursor abundance^3,14^. Chlorogenic acids represented the dominant phenolic fraction. The sum of 3-CGA, 5-CGA and 3,5-diCGA ranged between $1077 - 1125$ $\mathrm{mg}\cdot \mathrm{kg}^{-1}$ in Arabica and $1590 - 1694$ $\mathrm{mg} \cdot \mathrm{kg}^{-1}$ in Robusta samples. The higher levels in C. canephora agree with literature data reporting up to twice the total CGA content compared to C. arabica^13^. Among isomers, 5-CGA was the most abundant, confirming its thermodynamic stability under roasting conditions^24^. Ferulic acid, a degradation product of CGA, showed moderate differences among origins ($\approx 28 - 35$ $\mathrm{mg} \cdot \mathrm{kg}^{-1}$), indicating similar roasting intensities.

The profile of low-molecular-weight organic acids (citric, tartaric and acetic acids) revealed the expected transformation patterns associated with the roasting process. Citric acid, the most heat-labile component, decreased substantially compared to reported concentrations in green coffee (typically $4 - 6$ $\mathrm{g} \cdot \mathrm{kg}^{-1}$ in Arabica and $2 - 3$ $\mathrm{g} \cdot \mathrm{kg}^{-1}$ in Robusta^46^). In the roasted samples, residual levels ranged from $125 - 134$ $\mathrm{mg} \cdot \mathrm{kg}^{-1}$ in Arabica and $259 - 281$ $\mathrm{mg} \cdot \mathrm{kg}^{-1}$ in Robusta, reflecting degradation via decarboxylation. Conversely, acetic acid accumulated as a secondary product of carbohydrate pyrolysis and Maillard reactions, with higher concentrations in Robusta ($\approx 680$ $\mathrm{mg} \cdot \mathrm{kg}^{-1}$) than Arabica ($\approx 430$ $\mathrm{mg} \cdot \mathrm{kg}^{-1}$). Tartaric acid, known for its greater thermal stability, remained nearly constant across origins ($\approx 40-44$ $\mathrm{mg} \cdot \mathrm{kg}^{-1}$). The overall acid profile indicates that the higher acetic acid concentrations observed in Robusta coffees are consistent with their distinct chemical composition and roasting behaviour. Acetic acid formation during roasting is primarily associated with carbohydrate degradation, Maillard reactions and pyrolytic pathways; rather than with chlorogenic acid decomposition. Therefore, the observed differences are more plausibly related to variations in carbohydrate content and thermal reaction pathways between Coffea arabica and Coffea canephora, rather than to CGA levels alone.

The lipid yield for each sample was calculated based on an initial mass of 5 g per sample. The total lipid content ranged from 7.1% to 12.3%, with higher levels in Arabica than Robusta, consistent with previous compositional reports^9^. Lipids in roasted coffee are mainly triglycerides, diterpenes and free fatty acids. These are mainly generated through lipid-related processes, including triglyceride hydrolysis and thermal degradation occurring during roasting. These reactions may be promoted by high temperatures and moisture release within the coffee bean matrix, leading to the partial cleavage of ester bonds in acylglycerols and the subsequent release of free fatty acids. Their abundance plays a critical role in the retention of hydrophobic aroma compounds and the formation of crema in espresso beverages. The relatively high lipid recovery in the Arabica samples ($\approx 11-12\%$) supports their smoother mouthfeel and aromatic richness, whereas the lower lipid content in Robusta ($\approx 7-10\%$) is associated with greater bitterness and less body in the cup.

It is also worth noting that the experimental variability associated with lipid determination is higher than that observed for the other quantified compounds. This behaviour can be attributed to the gravimetric nature of the Soxhlet extraction method, which is inherently more sensitive to sample heterogeneity, solvent penetration efficiency and matrix-related effects compared to chromatographic techniques used for other analytes.

These trends are consistent with the known green coffee composition listed in Table 1, and the behaviour of the studied compounds during roasting, as described in Section "Behaviour of chemical compounds during roasting and modelling assumptions". To ensure consistency with the kinetic model, all measured concentrations are converted into a dry-weight basis ($\%\,w/w$) before the calibration procedure.

**Table 2:** Final composition of roasted coffee powders at the end of roasting. Values are reported as mean ± standard deviation.

Compound	Mexico (Arabica)	Rwanda (Arabica)	Nicaragua (Robusta)	Indonesia (Robusta)
Caffeine ($\textrm{CF}$) [mg/kg]	8491.40 ± 64.22	8615.33 ± 84.20	15258.71 ± 57.04	16607.39 ± 12.48
3,5-diCGA [mg/kg]	195.23 ± 1.21	201.66 ± 0.53	310.24 ± 1.18	329.10 ± 1.09
5-CGA [mg/kg]	675.62 ± 3.95	722.17 ± 3.27	980.82 ± 1.58	1050.11 ± 2.21
3-CGA [mg/kg]	206.34 ± 0.40	218.64 ± 1.61	300.11 ± 0.29	315.08 ± 1.83
Trigonelline ($\textrm{TR}$) [mg/kg]	321.67 ± 8.96	305.83 ± 7.26	227.18 ± 10.85	220.58 ± 10.55
Ferulic acid ($\textrm{FA}$) [mg/kg]	28.35 ± 0.36	34.96 ± 2.49	32.31 ± 0.73	33.29 ± 2.42
Tartaric acid ($\textrm{TA}$) [mg/kg]	41.24 ± 0.95	40.00 ± 0.80	43.72 ± 1.55	42.18 ± 1.38
Citric acid ($\textrm{CA}$) [mg/kg]	134.41 ± 6.19	125.17 ± 4.1	258.96 ± 1.96	280.93 ± 3.56
Acetic acid ($\textrm{AA}$) [mg/kg]	403.66 ± 1.14	456.24 ± 4.53	671.21 ± 2.01	693.39 ± 7.04
Lipids ($\textrm{LP}$) [$\%\,w/w$]	11.00 ± 1.35	12.30 ± 1.84	7.10 ± 1.56	10.20 ± 2.83

Non-lipid compounds are expressed in mg/kg (ppm) of roasted coffee powder. Lipids are expressed in percentage as mass fractions on a dry-weight basis ($\%\,w/w$).

### Temperature profile

**Fig. 2:**
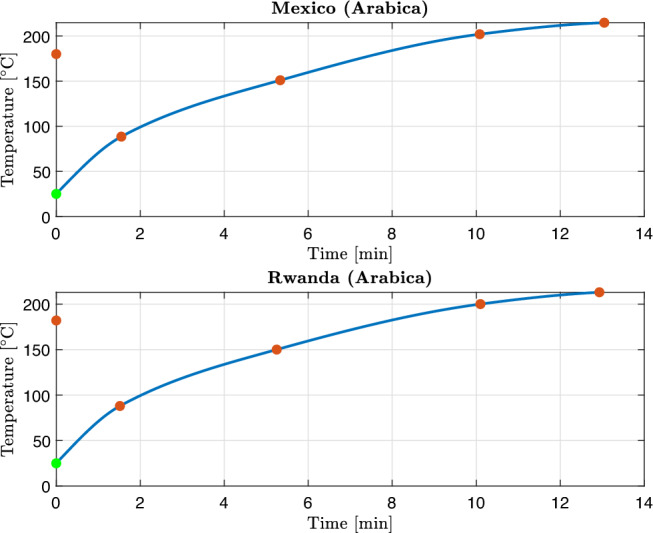
The blue solid curve represents the temperature profile $T_b(t)$ for the Arabica single-origin samples used in the numerical simulation. The red markers denote the roaster temperature measurements, and the green marker denotes the assumed initial temperature.

**Fig. 3:**
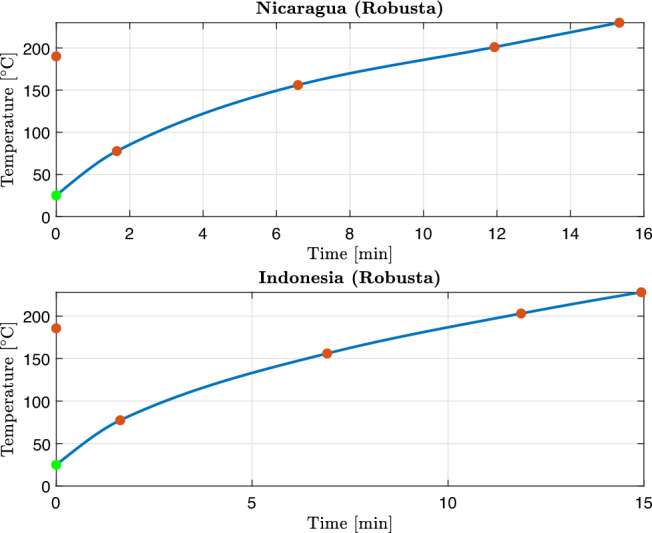
The blue solid curve represents the temperature profile $T_b(t)$ for the Robusta single-origin samples used in the numerical simulation. The red markers denote the roaster temperature measurements, and the green marker denotes the assumed initial temperature.

All four coffee samples were roasted on an industrial IMF drum roaster using standard production conditions. For each batch, Gambilongo Caffè srl provided the main temperature markers of the roasting curve: charge temperature, turning point, onset of the yellow phase, first crack and end-of-roast (drop) temperature. These markers are reported in Table 3. In the kinetic simulations, time is expressed in seconds, and the roasting temperature profile $T_b(t)$ for each sample is obtained as a continuous function by interpolation, and shown in Figures 2 and 3. This temperature profile $T_b(t)$ is used as a common input in the Arrhenius law for all reactions, hence no additional fitting of the thermal profile is introduced. In this way, the calibration procedure acts exclusively on the kinetic parameters, while the thermal history is prescribed by measurements.

**Table 3:** Roasting conditions and main temperature markers for the four coffee samples.

Marker	Mexico (Arabica)	Rwanda (Arabica)	Nicaragua (Robusta)	Indonesia (Robusta)
Charge temperature [*t*,${}^\circ$C]	0:00, 180	0:00, 182	0:00, 190	0:00, 185.6
Turning point [*t*, ${}^\circ$C]	1:33, 88.5	1:31, 88	1:39, 77.6	1:38, 77.4
Yellow phase [*t*, ${}^\circ$C]	5:20, 151	5:15, 150	6:35, 156	6:55, 156
First crack [*t*, ${}^\circ$C]	10:05, 202	10:06, 200	11:56, 201	11:52, 203
Drop/end of roast [*t*, ${}^\circ$C]	13:03, 214.8	12:56, 213	15:20, 230	14:56, 228

In detail, the curves in Figs. 2 and 3 show the roasting temperature profiles used in the numerical simulation for the four considered single-origin samples. In particular, the red points represent the temperature measurements obtained from a thermocouple positioned on the wall of the drum and reported in Table 3. We assume that the bean temperature starts at $25^\circ \textrm{C}$ (green point in Figs. 2 and 3), which is close to the ambient temperature, and then grows rapidly to reach the measured temperature. Hence, from the second red point onward, the four points are assumed to represent the temperature of the beans. Accordingly, the bean temperature profile $T_{b}(t)$ is obtained by a piecewise cubic Hermite interpolation of the five data points described above and shown in Figs. 2 and 3. In detail, the curves $T_b(t)$ are computed in MATLAB by means of the interp1 function, with the option ‘pchip’. Finally, we also emphasise that the choice of the first marker is an assumption that at least affects the early-time kinetics.

### Results of the kinetic model

We present the results of the calibration procedure for the proposed mathematical model for coffee roasting. First of all, we recall that we have introduced $\textrm{OTH}$, i.e. the fraction of other substances, to account for mass conservation. We notice that, also from a numerical point of view, the claimed mass conservation is reached within a tolerance of order $10^{-8}$, see Table 4. In this table, the maximum relative percentage error is listed for all the single-origin coffee samples; these values are obtained as

$$\begin{aligned} \max _t \left| \frac{M_{tot}(t) - M_{tot}(0)}{M_{tot}(0)}\right| \cdot 100. \end{aligned}$$

**Table 4:** Maximum relative percentage errors of the computed total mass *M*(*t*) during the ODE integration.

Blend	Variety	Relative percentage error
Mix Mexico	Arabica	1.1638e-09
Mix Rwanda	Arabica	2.5580e-12
Mix Nicaragua	Robusta	1.6311e-10
Mix Indonesia	Robusta	1.6257e-11

Relative percentage errors between measured values and simulated ones at the final time, i.e., at $t = \tau$, are listed in Table 5. As we can see, excellent agreement is obtained for the majority of the quantified chemical species among the single-origin samples. This suggests that the proposed calibration procedure is able to closely match the available end-of-roast chemical measurements under the considered conditions. However, it is also worth noting that in some of the single-origin samples, lipids exhibit the largest discrepancies, which is consistent with a higher experimental variability associated with gravimetric lipid determination. At this stage, these results should be interpreted as an assessment of the model’s flexibility to describe the roasting processes of coffee beans.

In Mix Mexico (Arabica), for most species, the relative error in percentage lies between $10^{-8}$ and $10^{-1}$, with the largest error for lipids. In Mix Rwanda (Arabica), the relative error in percentage at final time is less than $10^{-2}$, for all species, with the exception of lipids, which show an error of 5.79. In Mix Nicaragua (Robusta), most species show a relative error less than 0.20, while lipids show the maximum error. In Mix Indonesia (Robusta), errors are essentially zero; indeed, they are less than $10^{-8}$ for all species.

**Fig. 4:**
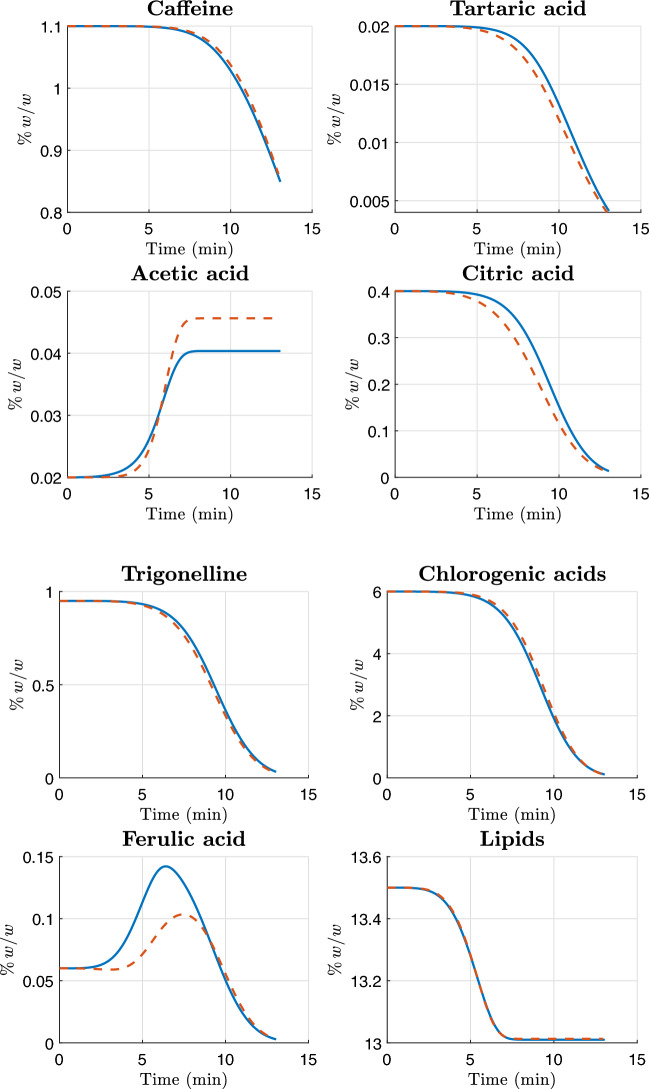
Concentration profiles of the measured compounds ($\textrm{CF}$, $\textrm{TA}$, $\textrm{AA}$, $\textrm{CA}$, $\textrm{TR}$, $\textrm{CGA}$, $\textrm{FA}$, $\textrm{LP}$) for the two Arabica single-origin samples. The solid blue line represents the evolution over time of the Mix Mexico single-origin coffee, and the dotted red line represents the evolution of the Mix Rwanda single-origin coffee.

**Fig. 5:**
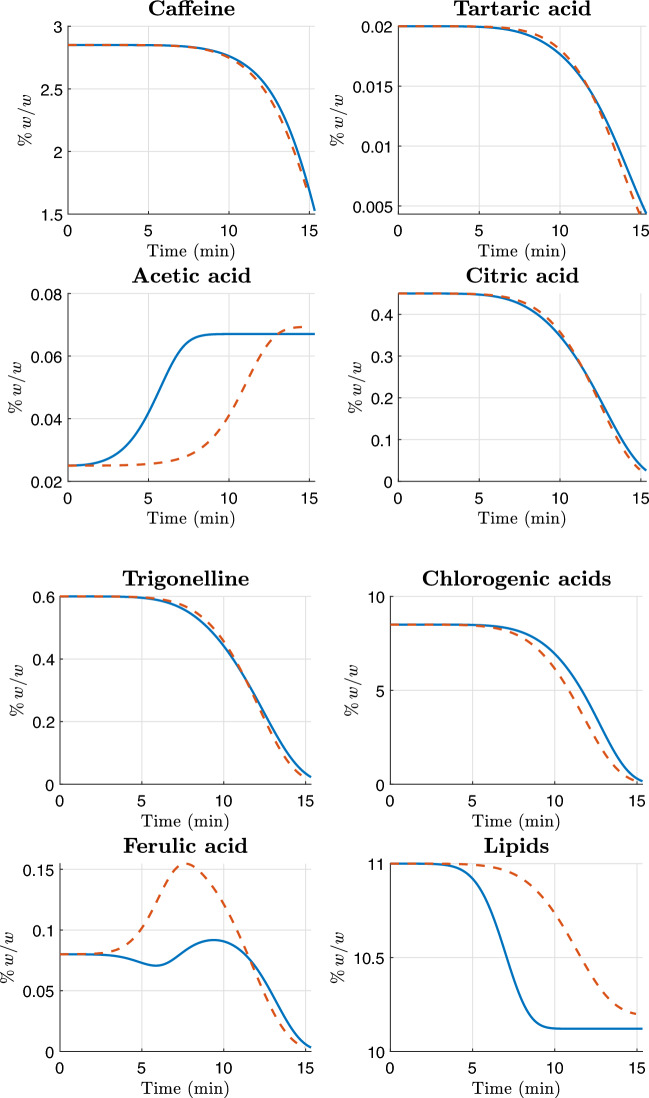
Concentration profiles of the measured compounds ($\textrm{CF}$, $\textrm{TA}$, $\textrm{AA}$, $\textrm{CA}$, $\textrm{TR}$, $\textrm{CGA}$, $\textrm{FA}$, $\textrm{LP}$) for the two Robusta single-origin samples. The solid blue line represents the evolution over time of the Mix Nicaragua single-origin coffee, and the dotted red line represents the evolution of the Mix Indonesia single-origin coffee.

The temporal profiles of the measured compounds are shown in Figs. 4 and 5. We remark that the intermediate-time concentration profiles shown are model-generated trajectories. In detail, the calibration procedure uses end-of-roast measurements, so that these intermediate-time trajectories should be interpreted as model-based predictions rather than experimentally confirmed kinetics. Across all four roasts, the model reproduces qualitatively reasonable trends for the tracked species. Caffeine, tartaric acid, citric acid, trigonelline and CGAs exhibit monotone decays compatible with an effective first-order behaviour. Acetic acid increases during roasting, while ferulic acid displays a formation-consumption pattern. Finally, lipids decrease smoothly. The corresponding optimal Arrhenius parameters $(k_i^{\textrm{ref}},E_{a,i})$ identified by the calibration are reported in Tables 6 and 7.

Within the Arabica panels, i.e. Fig. 4, the two single-origin samples show closely aligned trajectories for the most of the measured compounds, with only slight differences in the amplitude and/or decay rate. In the Robusta panels, i.e. Fig. 5, the profiles are more differentiated, particularly for ferulic acid and the CGA pool, consistently with their reactant-product relationship embedded in the mathematical model. Overall, the results indicate that this preliminary mathematical model for coffee roasting, driven by the measured temperature profile provided by Gambilongo Caffè srl, and calibrated only at the final time, can reproduce the end-of-roast concentrations with acceptable relative errors, listed in Table 5.

Moreover, the model yields qualitatively realistic temporal evolutions for the majority of the species. It should be noted that, since intermediate chemical measurements are not available, further experimental measurements during roasting will be necessary to confirm these dynamics and to enhance the predictive capabilities required for a digital twin.

**Table 5:** Relative percentage errors at final time for each measured species and single-origin coffee.

Species	Mexico (Arabica)	Rwanda (Arabica)	Nicaragua (Robusta)	Indonesia (Robusta)
Caffeine ($\textrm{CF}$)	7.2053e-08	8.9316e-08	5.0868e-07	8.6708e-12
Tartaric acid ($\textrm{TA}$)	3.0326e-08	1.2606e-07	1.9282e-05	4.8583e-11
Acetic acid ($\textrm{AA}$)	2.6011e-02	9.9023e-03	1.1727e-01	2.0092e-10
Citric acid ($\textrm{CA}$)	4.8324e-10	6.7523e-07	3.5136e-05	2.2082e-10
Trigonelline ($\textrm{TR}$)	8.5232e-11	6.3377e-07	2.4674e-05	3.0171e-10
Chlorogenic acids ($\textrm{CGA}$)	5.2831e-08	5.0630e-06	1.1017e-04	4.7222e-10
Ferulic acid ($\textrm{FA}$)	5.8922e-08	3.0091e-05	1.2714e-04	6.8991e-10
Lipids ($\textrm{LP}$)	1.8252e+01	5.7893e+00	4.2484e+01	2.8200e-12

**Table 6:** Optimal reference rate constants $k_i^{\textrm{ref}}$.

	Mexico (Arabica)	Rwanda (Arabica)	Nicaragua (Robusta)	Indonesia (Robusta)
$k^{\textrm{ref}}_{1}$	2.3763e-05	1.5166e-05	3.5328e-05	5.0141e-05
$k^{\textrm{ref}}_{2}$	1.4466e-04	2.8421e-04	1.7855e-04	1.4209e-04
$k^{\textrm{ref}}_{3}$	1.0922e-05	9.1560e-04	7.9492e-05	1.1630e-04
$k^{\textrm{ref}}_{4}$	5.2319e-04	4.7929e-04	5.0132e-04	4.1395e-04
$k^{\textrm{ref}}_{5}$	1.1896e-06	1.9342e-06	1.4989e-06	3.1343e-05
$k^{\textrm{ref}}_{6}$	1.0151e-03	1.7889e-03	8.2930e-02	7.6982e-04
$k^{\textrm{ref}}_{7}$	3.7263e-05	6.2401e-04	8.8913e-06	2.6232e-05
$k^{\textrm{ref}}_{8}$	1.0000e-06	1.1517e-06	1.0000e-06	2.6444e-05
$k^{\textrm{ref}}_{9}$	9.4181e-04	1.4146e-05	1.3745e-02	3.8173e-04
$k^{\textrm{ref}}_{10}$	9.9865e-04	5.1406e-05	1.0000e-03	3.6369e-05
$k^{\textrm{ref}}_{11}$	4.2515e-04	9.0666e-04	3.8697e-04	3.5367e-04
$k^{\textrm{ref}}_{12}$	4.2419e-04	5.1567e-04	4.6803e-04	4.2106e-04
$k^{\textrm{ref}}_{13}$	5.0386e-04	3.6364e-04	2.2884e-04	4.9929e-04
$k^{\textrm{ref}}_{14}$	1.9548e-02	2.4444e-02	2.4683e-02	2.5734e-02
$k^{\textrm{ref}}_{15}$	1.0000e-03	9.5798e-04	7.9041e-04	5.0210e-05

**Table 7:** Optimal activation energies $E_{a,i}$.

	Mexico (Arabica)	Rwanda (Arabica)	Nicaragua (Robusta)	Indonesia (Robusta)
$E_{a,1}$	109.9181	124.6180	122.3157	109.9405
$E_{a,2}$	109.9752	90.8344	100.0190	109.9741
$E_{a,3}$	104.9629	87.8731	121.3410	104.9826
$E_{a,4}$	104.9773	112.6491	96.7950	105.0052
$E_{a,5}$	104.9766	115.2129	115.6815	105.0028
$E_{a,6}$	105.0229	105.7871	122.5968	105.0231
$E_{a,7}$	104.9297	120.9254	123.4736	104.9468
$E_{a,8}$	134.8449	145.8624	109.2285	135.0001
$E_{a,9}$	110.0308	126.8107	139.7457	110.0046
$E_{a,10}$	60.0827	51.7517	48.1179	59.9898
$E_{a,11}$	100.0092	77.5419	95.4996	100.0035
$E_{a,12}$	100.0092	96.2164	93.8043	100.0091
$E_{a,13}$	100.0018	112.1700	121.7768	99.9926
$E_{a,14}$	100.1420	93.3901	103.2257	100.1612
$E_{a,15}$	100.1980	105.9417	111.7874	100.0466

## Conclusion

In this work, we formulate a mathematical model to describe the temporal evolution of the main chemical compounds in coffee beans during roasting. Starting from the chemical evidence about precursor-product relationships, we develop a connected reaction network that includes caffeine, tartaric acid, acetic acid, citric acid, trigonelline, chlorogenic acids, ferulic acid, lipids, sucrose, glucose, fructose and free amino acids. Moreover, motivated by the need to account for the most important chemical reactions, we introduce a sink variable representing the fraction of other compounds. This network is translated into a system of ordinary differential equations with first- and second-order kinetics; furthermore, we take into account for mass conservation via the $\textrm{OTH}$ variable. The concentration dynamics depend on the reaction rate constants, which in our model are all temperature-dependent through the Arrhenius law. The Arrhenius law is expressed in terms of two parameters, which are unknown and therefore must be estimated. For this reason, we devise a calibration procedure to estimate these parameters through a non-linear least-squares minimisation problem. The calibration was performed by minimising the discrepancy between the values simulated at the final roasting time and those obtained from chemical analyses carried out on four different types of coffee single-origin samples, specifically two varieties of *Coffea arabica L.* (Mix Mexico and Mix Rwanda) and two of *Coffea canephora Pierre ex A. Froehner* (Mix Indonesia and Mix Nicaragua). The results show a certain consistency with the theoretical evolution of the substances considered, and they represent a starting point for the development of a more complete and effective digital twin for coffee roasting. Future work will focus on acquiring intermediate time measurements during roasting. In particular, coffee samples collected at set intervals throughout the roasting process will undergo chemical laboratory analyses to monitor the evolution of each substance of interest. This will help refine the temporal behaviour of the concentration profiles. Furthermore, a second important future goal concerns the progressive refinement of the fraction of other substances, by performing appropriate chemical analyses on additional species that are not currently considered but are equally important, especially from a taste and sensory perspective.

Finally, in this paper, we consider only the kinematic aspects of the roasting process, as a first step toward the development of a digital twin for industrial applications. Additional conductive and diffusive processes relevant to coffee roasting modelling will be incorporated in future developments.

## Data Availability

The data are available in the manuscript.
